# Are Machine Learning Methods the Future for Smoking Cessation Apps?

**DOI:** 10.3390/s21134254

**Published:** 2021-06-22

**Authors:** Maryam Abo-Tabik, Yael Benn, Nicholas Costen

**Affiliations:** 1 Department of Computing and Mathematics, Manchester Metropolitan University, Manchester M1 5GD, UK; maryam.a.abo-tabik@stu.mmu.ac.uk; 2 Department of Psychology, Manchester Metropolitan University, Manchester M15 6GX, UK

**Keywords:** machine learning, deep learning, mobile computing, smoking, smoking cessation, smoking cessation apps

## Abstract

Smoking cessation apps provide efficient, low-cost and accessible support to smokers who are trying to quit smoking. This article focuses on how up-to-date machine learning algorithms, combined with the improvement of mobile phone technology, can enhance our understanding of smoking behaviour and support the development of advanced smoking cessation apps. In particular, we focus on the pros and cons of existing approaches that have been used in the design of smoking cessation apps to date, highlighting the need to improve the performance of these apps by minimizing reliance on self-reporting of environmental conditions (e.g., location), craving status and/or smoking events as a method of data collection. Lastly, we propose that making use of more advanced machine learning methods while enabling the processing of information about the user’s circumstances in real time is likely to result in dramatic improvement in our understanding of smoking behaviour, while also increasing the effectiveness and ease-of-use of smoking cessation apps, by enabling the provision of timely, targeted and personalised intervention.

## 1. Introduction

Smoking is considered a major public health problem with over 1.3 billion smokers worldwide [1]. Globally, in 2018, around 55% of smokers in developed countries considered quitting, but despite the availability of many treatments for nicotine addiction (e.g., support groups, nicotine patches, nicotine gum, etc.), less than one in ten smokers succeeded in quitting each year [2], suggesting that behaviour change, rather than dependence, may be the core difficulty in quitting.

Nicotine craving is considered the strongest motivator for smoking [3]; however, external factors such as time of day, location, alcohol consumption or the people with whom the smoker often smokes [4,5] can also stimulate the urge to smoke. This can be attributed to the repeated pairing of smoking with such external factors or events [6].

To understand how these external factors affect smoking, Shiffman et al. [7] used data from 406 smokers, split between daily smokers and intermediate smokers, who reported each cigarette and the related situation in an electronic diary. The findings suggested that while smoking patterns varied across days of the week and times of the day, similarities between smokers also emerged. For example, there were significantly more smoking events on the last working day of the week compared to other days, and smoking events generally peaked in the morning, stabilised in the afternoon and evening and slightly increased at night. Additionally, there was an association between smoking events and location, such that smokers were more likely to smoke when they were at home or in bars, but less likely to smoke when they were at work. Smokers’ activities also influenced smoking patterns, such that smokers in this study (who were by and large Americans) were less likely to smoke when they were working, even if the work was home chores. However, they were more likely to associate smoking with eating and drinking. On the other hand, leisure activities (i.e., hobbies, media consumption, sports, etc.) did not significantly affect smoking behaviour, except for media consumption, which resulted in an increase of smoking events among daily smokers, while having no effect on intermediate smokers’ behaviour.

This strong association between external factors and smoking suggests that during quit attempts, cravings are likely to be triggered by such associations [8]. This is confirmed by Dunbar et al. [3], who reported that a high level of craving was associated with situations where smokers had previously smoked. In turn, this suggests that while the nicotine level may be a strong predictor of smoking events while regularly smoking [9], associations formed with external cues such as locations, the people one used to smoke with or times of the day may become increasingly important following quitting. Therefore, to improve quitting rates, it is important to understand the smoker’s individual behaviour [6] and provide interventions that are timely and targeted to specific risk factors that are likely to trigger cravings in the smoker [4].

Smoking cessation mobile apps provide the perfect platform for such interventions. Mobile phones are regularly carried by most adults worldwide [10]. Typical smartphones are equipped with multiple sensors that are able to collect information such as the user location (Global Positioning System, GPS), identify other people who are near the user (by detecting Bluetooth signals), identifying users’ movements (walking/driving, etc., via onboard accelerometers) and potentially also additional information (such as specific arm movements), if connected to other devices such as smartwatches, which have also shown an accelerating increase in use around the world [11]. Combining this information with smoking events can enable the characterisation of smoking behaviours to be generated for individual smokers [12].

Integrating this information into smoking cessation apps can provide the data needed for “just in time” intervention [13,14], and improve the user’s experience [15,16]. Furthermore, since smartphones are now widely used by adults, they can provide easy-to-use, low-cost support to smokers (e.g., using apps, SMS, phone calls, etc.), in particular to groups that have been considered “hard to reach” using traditional methods (e.g., support groups, smoking cessation clinics) [17]. The efficiency of mobile health (mHealth) services has been proven in a wide range of fields, such as eating disorders [15,18,19] and pain management [20]. It has also been studied in the context of insomnia [16,21], stress management [22] and smoking [4,23].

This article provides an overview of up-to-date methods and techniques that have been used to implement smoking cessation apps, highlights the gaps in current approaches and suggests methods that can improve interventions and users’ experience by making better use of advances in mobile technology and machine learning models.

## 2. Common Approaches to the Development of of Smoking Cessation Apps

Smoking cessation apps can provide support to smokers in several ways. For example: they can provide advice on following better lifestyle routines, provide rewards for passing quitting milestones or support for the user in resisting the smoking craving during cessation [24,25]. However, while such support can be also provided offline via self-help books or seminars, the main advantage of smoking cessation apps is their potential to provide smokers with real-time, targeted support at a low cost [26].

As with other health-related apps, the smoking cessation app market has seen growth, but only a few of the apps have been scientifically examined or supported with randomised controlled trials that have assessed their efficacy [27,28,29]. An early approach in designing smoking cessation apps was very simple and contained self-reported trackers (e.g., money spent or cigarettes smoked) and smoking advice, which the user could access when logging in to the app. These apps rely entirely on the user logging their progress in the app and reporting their smoking relapses. Despite the simplicity and lack of scientific backing, these apps were shown to be effective in supporting smokers through the quitting process [30].

A more advanced approach to app-based interventions has been to shift from smoker-led to app-led interaction. In these apps, intervention messages are sent to the user at either periodic intervals or random times [31,32]. These messages have sometimes been personalised to the user, which usually requires that the users complete a demographic form and report preferences and smoking history, which are then used to select intervention messages. Examples include using the name of the user in the message or suggesting healthier life routines if the smoker had reported this as a motivation for quitting smoking [33,34]. While such apps have resulted in better user engagement compared with the previous approach [34], they still rely heavily on user input and are unable to make use of factors that influence craving and that are unique to the user.

Targeted intervention apps are based on the principle of identifying factors that influence craving and sending support messages at times of high risk, to help prevent relapse. Such apps can automatically identify the key moments (e.g., using GPS to identify a location where the smoker frequently smoked), by utilising a wide range of technological advances. Crucially, only a small proportion of the available apps currently make use of the full features of smart mobile devices [17]. There are thus many possibilities for improving the design and features of these apps so that they link better with real-time data, scientific evidence on smoking behaviour and also clinical outcomes [35].

An effective method for recording indicator variables that are associated with smoking events (and hence, enabling better targeting of interventions) is Ecological Momentary Assessment (EMA). This technique enables the collection of data in real-time and in their natural environment [36], and it has been used successfully to link smoking events with environmental information from smokers [37,38]. EMA has been employed in several smoking cessation apps, exploring the possibility of delivering interventions following the reporting of high-risk events for smoking such as alcohol consumption [39] or temporarily increased stress levels [40].

EMA was used by Businelle et al. [4] to examine the likelihood of predicting smoking relapse based on six common factors associated with smoking cessation lapse. The factors were: urge to smoke; stress; recent alcohol consumption; interaction with someone smoking; overall cessation motivation; and cigarette availability. Participants in the study self-reported their activities and smoking relapses, and this information automatically updated a diary located on their mobile phones. Results indicated that these factors provided a high level of information for predicting potential lapses. Similarly, Hébert et al. [37] collected relapse factors using EMA. Along with a weighting formula developed by Businelle et al. [4], the app estimated the risk of lapsing in real time and sent an intervention message that was either generic or tailored to the current level of risk of relapse. Results from both studies indicated that tailored messages were more effective than generic messages at preventing relapses.

Notably, one factor not used in these apps, but which has been found effective in predicting smoking events, is location [7]. Location may influence smokers in more than one way, in that smokers may associate specific locations with smoking (e.g., a specific spot outside the building where they work), but also, certain landmarks may trigger the urge to smoke. For example, Kirchner et al. [41] used EMA to examine the association between location and craving level. The study asked smokers during the first month of the quitting process to report their locations and craving level. Reporting was performed at regular or random intervals, prompted by the user or app as required. The researchers combined the reported data with tobacco sale locations, showing that exposure to tobacco sale locations was significantly related to lapses, even when the craving level was low.

However, while EMA apps are more efficient than earlier versions, for example, they typically make use of trackers [24], most of the available apps use self-reporting as a method to report craving factors or relapse events, which is an unreliable approach [4]. Long-term self-reporting is in particular likely to be affected by the “Ostrich problem” [42], by which people avoid monitoring their behaviour, as it may be tiresome or unpleasant or they may not be entirely committed to changing their behaviour. Therefore, data collection from mobile sensors can reduce the reliance on self-reports and increase the reliability of the smoking cessation apps [23]. By using mobile phone sensors (e.g., GPS, Bluetooth), smoking cessation apps can minimise user input, while providing more personal, targeted and, hence, effective interventions.

## 3. The Use of Passive Data Collection in Smoking Cessation Apps

In recent years, there have been significant improvements in the functionality of mobile phones; this improvement includes the addition of different built-in sensors (e.g., GPS, accelerometer, light). Smartphone sensors can be generally categorised as either motion or environmental sensors [43]. Motion sensors are used to measure the direction or speed of movement of the device. For example, the accelerometer is used to measure the magnitude and direction of acceleration forces applied over the smartphone on three axes, measured in meters per second squared (m/s${}^{2}$), and the gyroscope reflects the rotation rates on three axes in degrees per second (rad/s) [44].

On the other hand, environmental sensors are those that are used to measure different properties of the environment around the smartphone. This may include a light sensor to measure the intensity of ambient light, which is commonly used to adjust the brightness of the screen [45], and the microphone, which can be used for capturing environmental sounds [46]. Other sensors, such as the GPS, may be classified as sensors that capture both the environment and movement. The GPS sensor is used to track location coordinates (i.e., longitude, latitude and altitude), which may provide information about the environment, while also capturing the device’s speed. Longitude and latitude can reflect the location of the device in degrees, while the bearing is the horizontal travel direction, which is measured between zero and three-hundred sixty degrees. Speed refers to the rate of movement of the smartphone [47].

Combined, these sensors can be used to track and characterise the users’ motion and behaviours. The use of such sensors has been shown to be useful in different mobile applications, including games and health-monitoring apps (e.g., step counting) [48,49].

A systematic review by Cornet and Holden [50] considered 35 studies that used smartphone-based passive sensing for health applications. The review found that while most of the studies combined multiple sensors or built-in devices, the accelerometer was the most frequently used sensor (used by 25 studies for user activity recognition), followed by GPS (used in 22 studies). Other sensors or devices were less commonly employed, for example the light sensor (ten studies) or microphone (nine studies). Beside sensor data, some apps made use of analytical data including the call log, device on/off status and Short Message Service (SMS) patterns. The review highlighted the importance of using passive data collection in mHealth applications, in order to minimise the reliance on self-reporting by users and to increase the potential for providing just-in-time intervention using advanced analytic approaches.

While the use of passively collected data from sensors has revealed promising results in mHealth applications [51], there are limitations and ethical concerns that are related to passive data collection using a smartphone. One major concern relates to the privacy of participants’ data due to vulnerabilities in the security of data storage and, hence, potential unauthorised third-party access. This is of particular concern as mHealth apps that make use of passive data collection often collect sensitive information, such as demographic status, location tracking, and so on. This is of particular importance, as some behaviours targeted by mHealth apps may be socially or legally problematic. Besides data protection, other challenges have been raised such as high battery consumption [51,52]. These limitations and concerns are justified, and much work is invested into suggested solutions. For example, several studies have looked into data anonymisation (to minimise the risk of data uniquely identifying participants) and encryption (to ensure data security) [53,54]. Furthermore, to avoid battery consumption problems, some groups have considered using reduced sampling rates [55,56].

Passive sensing has previously been used to characterise and predict other addictive behaviours, such as alcohol addiction [57,58,59,60]. As an addiction, smoking is another behaviour that shows potential to improve intervention by combining mHealth applications with passive sensing. Smoking cessation apps have thus seen a shift towards combining EMA with passive data collection.

One such attempt toward passive data collection was a feasibility study undertaken by Naughton et al. [23]. The app in this study delivered automated targeted messages, based on auto-collected geolocation data. The study collected data from 15 participants over a two-week pre-quit period and then for 28 days after the participant-set quitting date. The QSense app sent notification messages when the participant entered an area previously identified by the participant to be of “high risk”, meaning locations where they used to smoke. High-risk areas were identified when the smoker logged smoking events in particular locations during a two-week pre-quit smoking period. After the quitting date, the app automatically identified the high-risk location when the person stayed in a previously saved location for at least five minutes. Results suggested that passive data collection and, in particular, geolocation detection could significantly assist in providing targeted intervention. Following the 28-day post-quitting period, only 50% of the participants reported relapse events with an average of total three lapse events per participant; the first lapse happened on average on the ninth day of smoking absence. This suggests that understanding the individual’s smoking patterns and delivering intervention messages at critical times based on the individual’s needs can improve the smoking cessation success rate.

Given that simply using the location improved cessation outcomes, producing more sophisticated models that are individually tailored and include more than one craving factor is likely to improve outcomes further. This requires the use of advanced analysis tools such as machine learning in order to enable effective and efficient processing of the volume and complexity of the data. In addition to passive data collection of this type, it is perhaps important to provide distractions or activity suggestions. For example, the GPS could be used to identify attractions that are near the current location that may distract from smoking (“don’t smoke, you can do something else...”). However, it has not been possible to find any studies that have explored this option.

## 4. Machine Learning Methods for Auto Intervention: The Future of Smoking Cessation Apps

Machine Learning (ML) methods have been employed to perform tasks efficiently without human guidance. ML problems are mainly classified into two categories: classification problems and regression problems. In classification problems, the prediction model’s task is to assign the input samples to a certain category. In regression problems, the input samples are used to generate continuous data [61]. The design of ML models can be either supervised or unsupervised. In supervised learning, dependent variables are predicted from independent variables by a mapping function. The dependent values in the training set are predetermined, by using other sources such as the previous literature and the judgement of field experts or users to annotate the samples. In contrast, unsupervised learning does not require a specific outcome variable, but clusters observations simply on the independent variables. In all ML models, the model parameters are tuned using a portion of the dataset; this is called the training process. Then, the model performance is tested using the remaining (i.e., unseen part) of the dataset; this is the testing process. Often, part of the training section of the dataset is used solely to assess the suitability of the model’s parameters; this is referred to as evaluation. The resultant ML model is tested based on its prediction accuracy, which can be defined as its ability to identify relationships and patterns among input features in a previously unseen dataset [62].

ML has been shown to be an effective method for predicting several addictive behaviours in users, based on the processing of environmental data. Examples include gambling, alcohol use, cocaine use and smoking [63,64]. A systematic review by Mak et al. [64] identified that supervised learning methods (e.g., Classification Trees (CTs), naive Bayes, logistic regression, Support Vector Machines (SVM), neural networks) were used more often than unsupervised learning methods in these applications and have shown a better prediction accuracy. In fact, the review only identified two studies that applied unsupervised learning methods, and both used this method to classify a large set of participants into groups. Gray et al. [65] used k-means clustering algorithms to identify gamblers among 217 employees of a casino, and Sun et al. [66] used k-medoid clustering and hierarchical clustering to group a large sample of people (*n* = 5390) according to their opioid dependence symptoms, in order to extract a heritability component of opioid dependence. This suggests that there may be scope for future research to utilise such methods to identify different groups of smokers according to their smoking habits (e.g., number of cigarettes per day, years of smoking, starting age, etc.) in order to provide better targeted interventions.

An example of a ML algorithm that was developed to understand smokers’ behaviour and the urge to smoke as it changes during the quitting period was described by Koslovsky et al. [67]. The authors proposed the use of Bayesian structural time series as a robust method to analyse the risk factors associated with different stages of the quitting process. They demonstrated that feeding self-reported EMA data into Bayesian variable selection for multistate Markov model methods could be effective at modelling the transition between two smoking states (i.e., smoking, non-smoking). The study made use of data collected from one-hundred and forty-six participants (using EMA) over a period of two weeks. Twenty factors were collected four-times a day, at random intervals. Factors included information such as cigarette availability, location, urge to smoke and behavioural and environmental factors. The results were able to identify the association among these 20 factors and their importance during the transition from the smoking to non-smoking state, and from the non-smoking to smoking state, in order to inform future studies about what may be helpful in the design of effective, real-time smoking cessation interventions.

Several studies have identified CTs as a particularly effective method in smoking research [63,68,69,70,71]. CTs are a well-known ML algorithm that uses a tree structure to classify the data. To this end, the input features are partitioned at each node based on testing criteria, forming a branch, which is then further divided on the basis of other tests until it reaches a node that corresponds to the classification decision [72]. In CTs, each leaf represents a decision about the membership of a certain observation. The most common type of CT is known as CART [73], which uses a metric selection step based on information theory. A more advanced method of CTs is to use multiple CTs at the same time; this can help capture different classification rules. This type of CT is called a Decision Forest (DF), and it is a more suitable approach for solving complex problems [74].

An interesting attempt at using CTs was that of Dumortier et al. [68], who used historical data collected from university students to evaluate the urge to smoke based on 41 self-reported features (e.g., alcohol consumption, mood status, hunger, location, type of work, etc.). Comparing three different ML algorithms (naive Bayes classifier, discriminant analysis classifier, and CT), results revealed that ML had the ability to estimate smokers’ urge rating with, in general, a high accuracy and that the CT performed best with up to 86% accuracy. Furthermore, while the results indicated that the number of features used by the classifier could increase the accuracy, this can be computationally time consuming. Therefore, the study suggested the use of a spatial feature selection approach to minimise the model analysis time, while maintaining some level of prediction accuracy. Using this approach reduced the model accuracy to an average of 68% (when only four features were selected), with the specificity dropping from 50% to around 35%. This example of using ML to understand smokers’ behaviour, and how this can be used to predict potential lapses in real time, is encouraging. However, this study did not make use of passively collected data, and it is impossible to imagine smokers regularly reporting (up to) 41 features or risk factors. As such, data such as coffee consumption, mood, and some other combinations of self-reported information should be minimised, and more variables extracted from the environment automatically should be considered.

A recent and more advanced approach attempting to understand smokers’ behaviour is the use of Deep Learning (DL). DL involves multilayer artificial neural networks and has performed very well in many complex classification problems [75,76,77]. This high level of performance reflects the combination of a full cascade of non-linear transformations of the data, each specified in conjunction with the others, and the use of very large training sets, made possible by the use of non-closed-form training algorithms. One of the most common and successful DL modes is the Convolutional Neural Network (CNN) [78]; the CNN is designed as a sequence of convolutional layers, each of which uses sliding windows (kernels) and filters for feature extraction.

A study by Abo-Tabik et al. [79] modelled smoker’s behaviour using a 1D-CNN. The research compared three different supervised ML models (CT, SVM, and 1D-CNN) in capturing the individual differences in smoking behaviours, based on data collected from mobile phone sensors. The model was combined with the control theory description of smoking [9] to enable the system to capture both internal (i.e., nicotine craving) and external (location and motion) factors that could influence smokers’ behaviour. Three accelerometer (motion) and three GPS (location) values were collected every minute for two weeks from participants’ mobile phone sensors, while participants self-reported their smoking events by pressing a button on their home screen. Analysis using the three different ML models revealed that the 1D-CNN performed better than the other methods. The CNN model reported in the paper consisted of six parallel sub-models, each taking one of the six input features. Each sub-model has a convolutional layer with 64 filters, and all the sub-models were combined into a fully connected neural network. This gave a final output identifying the input as the behaviour leading up to a smoking or non-smoking period. The model achieved an overall accuracy of 86.6% when predicting a smoking event within a 30 min window. Furthermore, the findings suggested that both participants’ movement and their location contributed to the accuracy of the model, suggesting that the precision of prediction could potentially improve with more parameters. These parameters could include detecting an indoor/outdoor situation (using light sensors), or incorporating information about known locations (e.g., tobacco sales points, pubs), or personalised parameters such as stress level [63]).

There is an important limitation to the model suggested by Abo-Tabik et al. [79]: the app still required participants to self-report their smoking events during the pre-quit period. However, it has been shown that using ML on data collected using wearable devices (e.g., smartwatches) can reliably distinguish between smoking-related hand movements and other similar behaviours such as drinking, eating, brushing teeth, etc. [80]. It thus may be possible to ask participants to wear a smartwatch on their preferred smoking hand during a pre-quit period in order to capture smoking behaviour without self-reporting being needed. Alternatively, devices such as instrumented cigarette lighters might be used. Lastly, although the ML algorithm was able to predict smoking events while the user was still smoking, there remains a lack of understanding of craving behaviour and potential lapses after the smoker decides to quit. In other words, more work is required to understand what parameters need to be collected and how they should be modelled and used in order to send effective targeted messages.

While it is clear that ML, combined with up-to-date technological advances such as those provided by mobile phones and smartwatches, can provide a perfect platform for delivering targeted personalised and timely interventions [81,82], these methods suffer from the limitation that the smoker needs to have an extra wearable device besides their personal smartphone to predict smoking events.

In contrast, the study by Schick et al. [83] showed a first attempt at combing real-time data analysis using ML with targeted messages. Schick et al. [83] used a combination of EMA and ML to predict potential urges to smoke based on geolocation. The ML model in the app, a hidden Markov model, was trained on pre-quit data (i.e., the timing and places in which individuals were most likely to smoke). The app then used these patterns and real-time information to provide timely and context-relevant support messages during the quitting period. While the study did not report any quantitative results that were related to the hidden Markov models, some participants reported receiving messages at inappropriate times. These had the opposite (and undesirable) effect of reminding them about their smoking habit, highlighting the need for a very accurate level of modelling. However, overall, the feedback from participants was positive, suggesting that implementing smart apps to support users may not only be beneficial, but also improve users’ experience.

Based on previous studies, it can be suggested that using passive data collection combined with ML methods could improve the effectiveness of smoking cessation apps. While there have been good attempts at using these approaches, more work is required, including large-scale studies, in order to understand the feasibility of such an approach in providing real-time interventions. In addition, while the main aim of the studies described above was to find ways for employing ML methods to improve smoking cessation apps, it is important to consider that the type of study, collected data, and experimental settings differed across the different studies. This makes it impossible to reliably compare their outcomes. However, in general, work needs to be performed to minimise the reliance on unreliable self-reporting of craving factors and move toward passive data collection, as it has proven to be a more reliable and effective way to support intervention. Furthermore, while DL appears to result in better predictions of smokers’ behaviour, more work is required to improve the methods of data collection and the validation of smoking event reporting during the pre-quit period, in order to confirm this observation.

## 5. Limitations and Future Directions

While the literature to date presents very promising results on the possibility of employing ML to improve the development of smoking cessation apps, major challenges should be considered and addressed. In addition to the ethical and data security challenges that were mentioned above, there are several technical challenges that should be considered. First, given that the number of smoking event samples is much smaller than the non-smoking samples (i.e., in 24 h, there are 1440 min, and even if we consider relatively heavy smokers, who may smoke 20 cigarettes a day, if each cigarette takes 5 min, the person will only smoke for 100 out of the 1440 min per 24 h), the resultant datasets are strongly unbalanced. This problem can cause inaccurate classification prediction [84] and may result in bias in the the prediction of non-smoking events (i.e., if the algorithm predicts a non-smoking event for each of the 1440 min, it may possibly result in a 93% correct prediction).

Another important challenge for this type of research is the lack of published open-access datasets, which prevents systematic research on smoking behaviour. Hence, this makes it difficult to both analyse large samples and to compare results across different studies [79]. While the nature of the data (e.g., location or other identifiable data) is a concern for data sharing [51], these concerns have been addressed in many other difficult circumstances (e.g., medical data). Achieving a similar level of data sharing while ensuring participants’ data privacy is an essential step in developing this area of research. While the field follows the pattern of individual datasets collected by and for individual groups and studies, it will remain impossible to determine whether differences in performance shown by various algorithms reflect the algorithms themselves, the peculiarities of the data-collection procedures or some combination of these effects.

## 6. Conclusions

We have mapped the development of smoking cessation apps to date and outlined the need for a focus on smarter apps that make full use of available technology in order to address one of the biggest preventable killers in modern times: smoking. This article explored methods for developing smoking cessation apps, highlighting the need to improve our understanding of smokers’ craving factors, in particular as they change through the quitting period, in order to improve targeted and timely interventions. We further explored how data that enable the mapping of smoking and craving patterns may be collected (with or without user inputs), as well as the importance of sharing existing datasets. Lastly, we highlighted the likely improvements (to the quality of the data, the effectiveness of the intervention, and the user satisfaction) that could be achieved by minimising the use of self-reported craving factors and smoking behaviour, by increasing the reliance on passive data collection from smartphone sensors.

The evidence indicates that in order to improve the performance of smoking cessation apps, it is necessary to minimize the reliance on self-reported data, which could be achieved with greater use of passively collected data from different mobile sensors (GPS, accelerometer, gyroscope, etc.). Location data that are automatically collected using built-in GPS have already been tested and shown to be able to improve intervention effectiveness [23]. Combining location data with passively collected accelerometer data further improved the prediction of smoking events, which could lead to further improvement of targeted interventions. In terms of data analysis, DL appears to be better suited for the task of predicting smoking behaviour compared to other ML methods, and findings from studies using CNN report promising results in predicting smoking events [79]. Finally, the results from the papers reviewed suggested that future work should focus on improving the prediction performance of smoking behaviour. This could be achieved by selecting better prediction models including those that have proven their efficiency for time series data analysis, such as Long Short-Term Memory (LSTM) [85]. Such models have been previously used successfully for human behaviour recognition [86,87] and health applications [88]. Improvement could also be achieved by using better methods for selecting a near-optimal DL model architecture. For example, instead of using naive random searches for selecting a DL model’s hyper-parameters, other advanced approaches such as genetic algorithms [89] could be used. Combined with passively collected values from sensors such as light (for detecting of indoor/outdoor environments) or gyroscope (for improving motion recognition) sensors, this could add significant improvement to the prediction of smoking events. While we were not able to identify any studies exploring these techniques and data sources, we believe that advances of this sort could lead to much improved targeted and personalized interventions to help people overcome this dangerous, but hard-to-stop habit of smoking.

Overall, while it is clear that ML combined with up-to-date technology such as that provided by mobile phones and smartwatches can provide a perfect platform for delivering personalised and timely interventions, no apps to date have made full use of all of these techniques. Importantly, we have highlighted areas that research and research practices should improve, as well as the full range of available technology and the advanced ML algorithms that could be used to support smokers in the difficult task of quitting.

## Data Availability

Not applicable.
